# Experiment-Driven Atomistic Materials Modeling: A Case Study Combining X-Ray Photoelectron Spectroscopy and Machine Learning Potentials to Infer the Structure of Oxygen-Rich Amorphous Carbon

**DOI:** 10.1021/jacs.4c01897

**Published:** 2024-05-15

**Authors:** Tigany Zarrouk, Rina Ibragimova, Albert P. Bartók, Miguel A. Caro

**Affiliations:** † Department of Chemistry and Materials Science, Aalto University, Espoo 02150, Finland; ‡ Department of Physics, University of Warwick, Coventry CV4 7AL, U.K.; § Warwick Centre for Predictive Modelling, School of Engineering, University of Warwick, Coventry CV4 7AL, U.K.

## Abstract

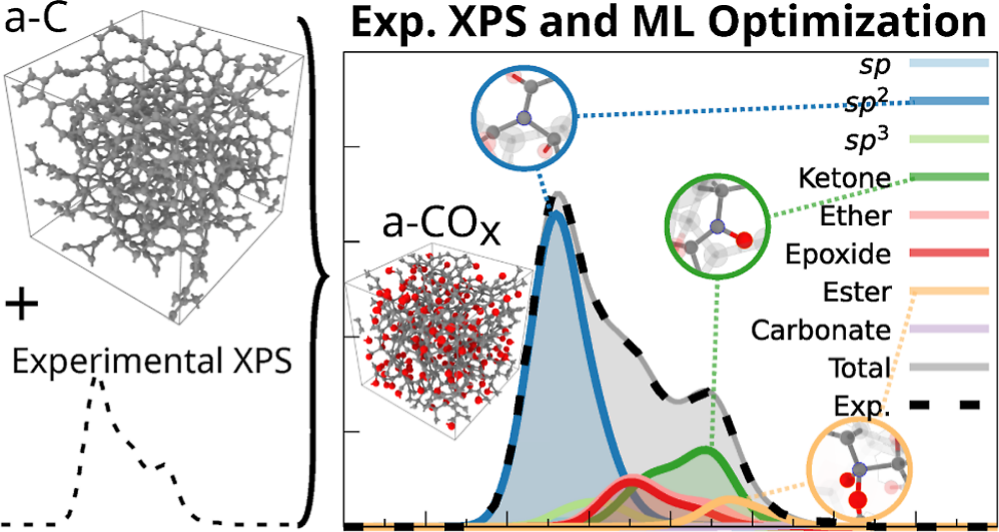

An important yet challenging aspect of atomistic materials modeling is reconciling experimental and computational results. Conventional approaches involve generating numerous configurations through molecular dynamics or Monte Carlo structure optimization and selecting the one with the closest match to experiment. However, this inefficient process is not guaranteed to succeed. We introduce a general method to combine atomistic machine learning (ML) with experimental observables that produces atomistic structures compatible with experiment *by design*. We use this approach in combination with grand-canonical Monte Carlo within a modified Hamiltonian formalism, to generate configurations that agree with experimental data and are chemically sound (low in energy). We apply our approach to understand the atomistic structure of oxygenated amorphous carbon (a-CO_*x*_), an intriguing carbon-based material, to answer the question of how much oxygen can be added to carbon before it fully decomposes into CO and CO_2_. Utilizing an ML-based X-ray photoelectron spectroscopy (XPS) model trained from *GW* and density functional theory (DFT) data, in conjunction with an ML interatomic potential, we identify a-CO_*x*_ structures compliant with experimental XPS predictions that are also energetically favorable with respect to DFT. Employing a network analysis, we accurately deconvolve the XPS spectrum into motif contributions, both revealing the inaccuracies inherent to experimental XPS interpretation and granting us atomistic insight into the structure of a-CO_*x*_. This method generalizes to multiple experimental observables and allows for the elucidation of the atomistic structure of materials directly from experimental data, thereby enabling experiment-driven materials modeling with a degree of realism previously out of reach.

## 1 Introduction

One of the objectives of computational materials modeling is to infer the atomistic structure of matter, both for the purpose of satisfying our curiosity about what matter “looks like” at the atomic scale, and to obtain structure–property relations which might help in the design of useful materials and molecules. However, real materials often have a complex structure, which might set the time and/or length scales required for accurate simulation beyond the reach of density-functional theory (DFT). Fortunately, in recent years, data-driven approaches that “learn” the DFT potential energy surface (PES) with high fidelity have been developed. These so-called machine learning (ML) potentials (MLPs) use ML-based techniques such as artificial neural networks^1,2^ or Gaussian process regression (GPR)^3,4^ [and the related kernel ridge regression (KRR) method] to make DFT-quality predictions of energy and forces for a small fraction of the CPU cost.^5−8^

Thanks to the flexibility of these ML approaches, properties other than energies and forces can also be predicted from the atomic structure. Recent examples include electron density^9,10^ (or even wave functions^11^), atomic charges,^12−14^ molecular dipoles,^15,16^ adsorption energies,^17,18^ spectroscopic signatures,^19^ and X-ray spectroscopy in particular.^20−27^ Some of these predictions, specifically spectroscopic calculations, are amenable to direct comparison with experiment. This opens the door to designing new ways to sample a material’s configuration space, combining a computationally cheap and accurate model of the PES with improved recipes for how to navigate it. This has the potential to provide a tight integration between experimental data and simulation,^28−30^ leading to a paradigm shift in materials modeling and atomistic structure prediction. This new paradigm, that we call “experiment-driven atomistic materials modeling”, is particularly relevant to improve our understanding of complex and amorphous materials.

In this paper, we focus on the integration of *ab initio*-accurate MLPs and ML-based computational X-ray photoelectron spectroscopy (XPS) models to generate low-energy structures which also have experimental XPS agreement. First, we use classical atomistic simulation methods based on molecular dynamics (MD) and Monte Carlo (MC) to sample the MLP PES and show that the resultant XPS predictions of final structures do not agree well with experimental XPS spectra. Then, we detail a method to combine these models, by performing an on-the-fly prediction and validation against the experimental reference of the XPS during structural optimization with MC. This “generalized Hamiltonian” or “modified dynamics” formalism results in atomistic structural models that produce the same observables as the experiment *by design*, while ensuring the models remain structurally and energetically sound.

We present this method together with a case study concerning the elucidation of the atomistic structure of oxygen-rich amorphous carbon (a-CO_*x*_). This material has potential applications such as in memristors,^31,32^ and by varying the degree of oxygenation, the tribological and electrochemical properties of oxygen-free a-C^33,34^ could be tuned. Indeed, understanding a-C oxidation is key to predict and potentially mitigate the long-term effects of friction on a-C coatings.^35^ An important fundamental question pertaining to a-CO_*x*_ is also of interest: how much oxygen can be added to carbon before it inevitably burns? Although we focus here on XPS as our experimental target, because it is an experimental technique widely used for the study of disordered materials, the methodology and approach are general and extension to other experimental techniques is straightforward. Our group is in active development with regards to these extensions.

## 2 Interpretation of XPS

XPS is a ubiquitous experimental method used to measure the distribution of core–electron binding energies (CEBEs) in a material. A specimen is irradiated with monochromatic X-rays which excite core electrons such that they are photoemitted. The difference in the measured kinetic energy of these electrons and the energy of the incident X-ray gives a range of CEBEs. The usefulness of this method stems from the fact that the binding energy of a core electron depends on its local environment, allowing for inference of the structure of a material upon inspection of its XPS spectrum. CEBEs of reference environments—say an sp^2^ carbon atom in graphite or a molecule where carbon is bonded to a certain functional group—can be measured. Choosing a number of reference environments which one expects in the material, and assuming that environments in the material similar to those of the references give similar CEBEs, one can approximately determine the composition of an unknown structure by *deconvolving* the measured spectrum. Deconvolution splits a spectrum into peaks, centered at the reference CEBEs, which sum to give the measured spectrum. The ratio of peak areas gives the relative proportion of different environments. In this section, we highlight some of the issues with this approach and outline how simulations may help to overcome them.

### 2.1 Reference Peak Energies are not Transferable

We examine the validity of reference peak values commonly used in XPS peak fitting^36^ with an example from the literature. The issue of peak assignment and practicalities of fitting XPS curves has already been discussed by others (see, e.g., refs (37–39)). We propose an alternative method for fitting in Section 4.6 and show results in Section 5.3.

**Figure 1:**
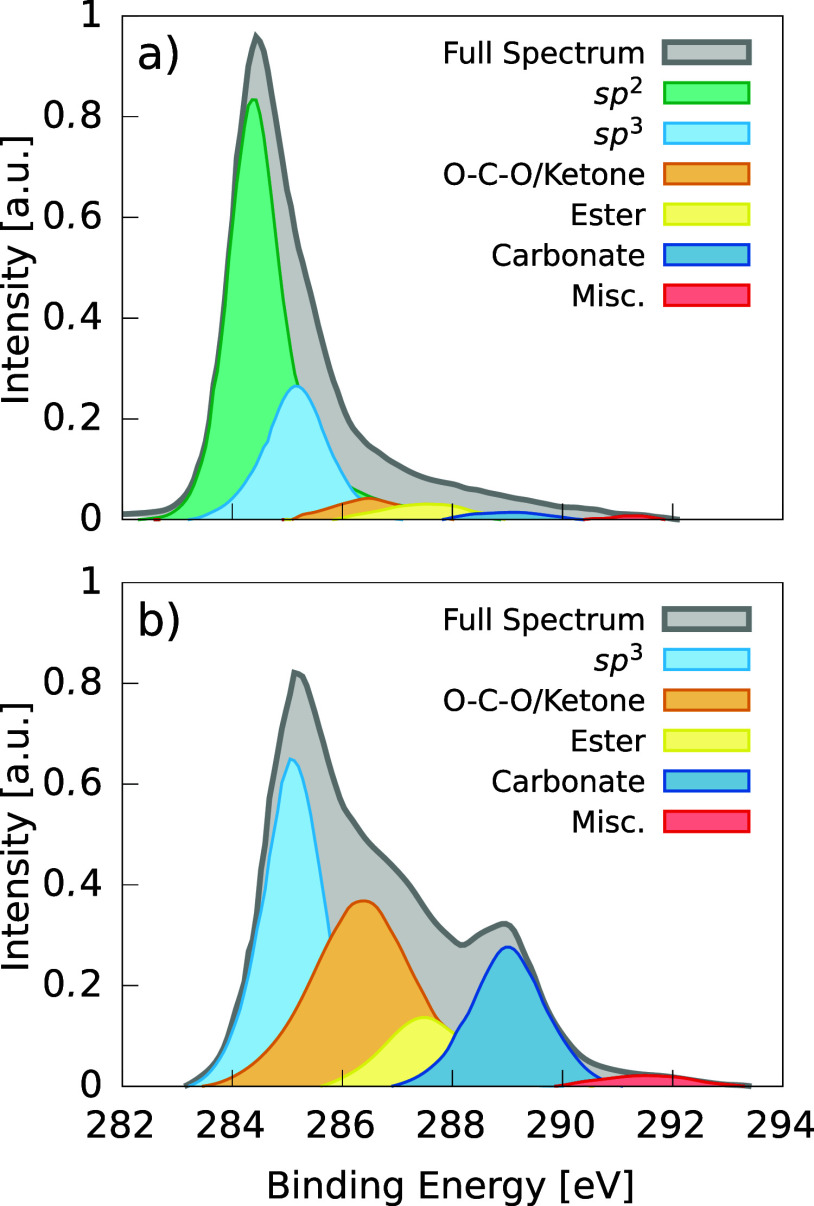
Example of experimental C 1s XPS deconvolution of (a) an a-C structure (reported O/C ratio 0.1) and (b) a-CO_*x*_ (reported O/C ratio 0.6) performed by Santini et al.,^31^ including the peak assignment proposed by the authors. We have subtracted the background by the method of Shirley.^40^ It can be shown within our simulation framework that the whole spectrum of (b) has been shifted toward larger C 1s binding energies due to the presence of oxygen atoms, which shift the electrostatic potential in their immediate surroundings. This shift does not allow for sp^2^ motifs to be detected by the deconvolution algorithm as the sp^2^ reference is fixed.

We are interested in elucidating the structure of oxygen-containing carbon materials, hence we inspect the work by Santini et al.^31^ on a-CO_*x*_, which was also examined in our previous work.^24^Figure 1, replotted from the original results, shows their peak assignment based on standard reference values for a-C and a-CO_*x*_. The overall XPS C 1s spectrum is reconstructed by the sum of its contributions. These contributions are from sp^2^ carbons, sp^3^ carbons, and carbons which are part of different O-containing functional groups. We will use Figure 1 to illustrate one of the pathological issues associated with traditional XPS peak fitting, namely that standard references established for one class of materials are not transferable to other (possibly more complex) materials.

**Figure 2:**
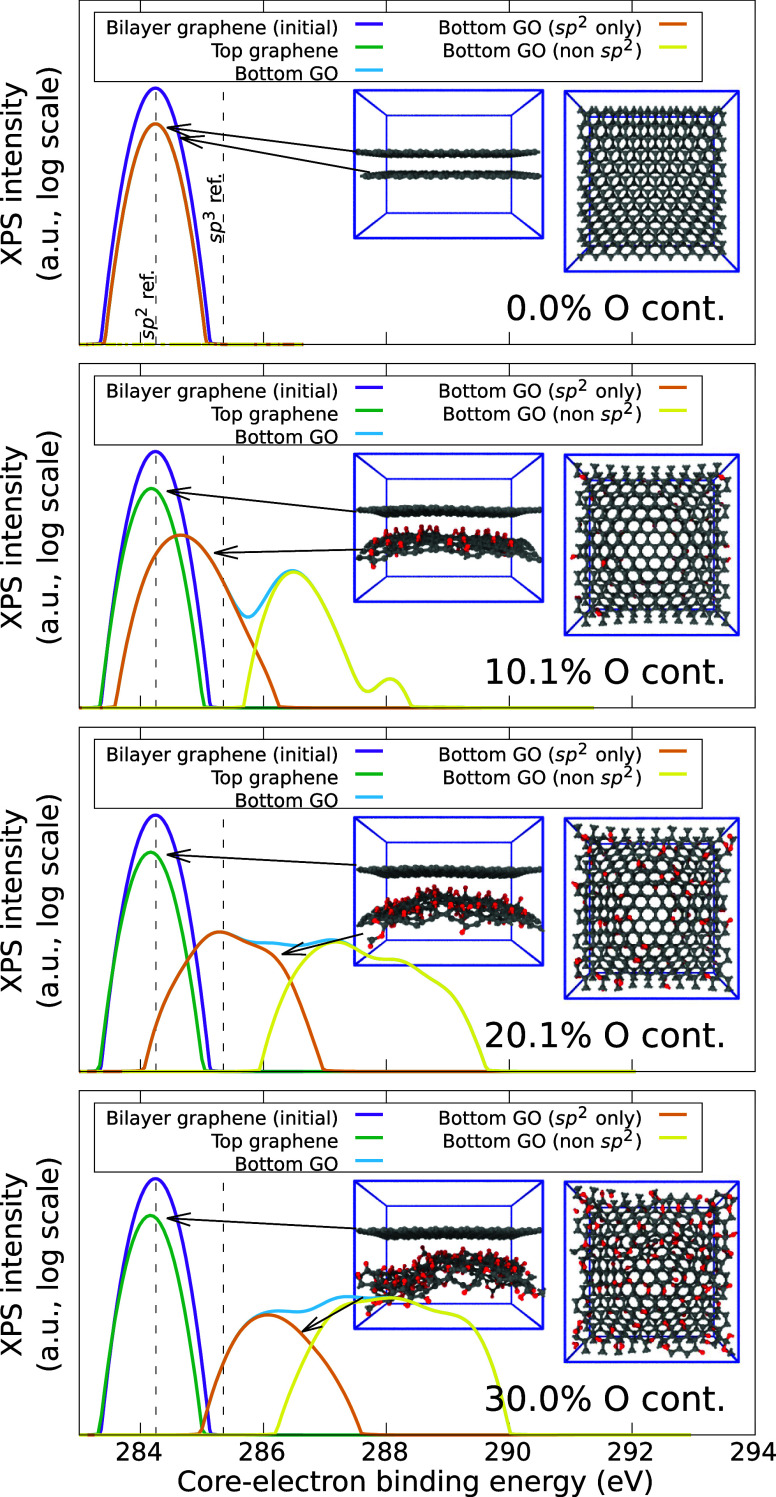
Simple computational experiment to illustrate how the sp^2^ C 1s peak shifts to higher CEBEs as the amount of oxygen increases. The bottom layer in the initial bilayer graphene structure (top panel) is progressively oxidized toward high O content (approximately 10% increase with each panel moving downward in the graph) and its sp^2^ contribution to the XPS spectrum shifts accordingly.

Let us consider the following computational experiment, whose results are depicted in Figure 2. We start out with a bilayer graphene system, where the predicted XPS spectrum can be trivially split into the contribution of the upper layer and that of the bottom layer, since our ML CEBE model^24^ predicts individual CEBEs which are unambiguously assigned to individual atoms. Initially, the contributions to the overall spectrum from top and bottom layers are identical. We proceed to add oxygen to the bottom layer in the form of epoxide functional groups. These groups are only introduced with the oxygen pointing from the bottom layer toward the middle of the bilayer system so that possible interactions with the top layer can be easily detected. To avoid generating unphysical structures, which could introduce artifacts in the predicted XPS spectrum, we proceed according to a *heuristic* approach inspired by Markov-chain grand-canonical MC (GCMC), where the potential energy of the system is described with the MLP introduced in Section 4.1. For context, we mark the sp^2^ reference peak position at 284.25 eV and the sp^3^ reference at 285.35 eV, i.e., the difference being 1.1 eV which is the commonly accepted splitting between sp^2^ and sp^3^ peaks in a-C.^41^ We further split the XPS spectra into the C 1s contributions from sp^2^ motifs, defined as any given C atom bonded exclusively to three other C atoms, and C atoms bonded to any number of neighbors one or more of which is an O atom (labeled as “non sp^2^” on Figure 2). As more oxygen is incorporated into the resulting graphene oxide (GO) structure, we monitor the evolution of the bottom and top XPS spectra.

We can clearly see three effects: (1) the CEBEs of the top sp^2^ C atoms largely remain at the reference value, this is what is expected according to conventional XPS peak fitting wisdom; (2) the C 1s levels corresponding to C atoms directly bonded to O atoms have significantly larger binding energies than pure carbon motifs, also in accordance with conventional wisdom; (3) there is a very strong shift of the sp^2^ peak in the bottom layer toward higher energies due to the presence of O, even though the sp^2^ C atoms remain bonded only to other C atoms. At high-enough O content (∼20%), this shifted sp^2^ peak directly overlaps with the *reference* sp^3^ peak and, at even higher O contents, it shifts to values significantly higher than the sp^3^ reference. This conflicts with the common practice of using immutable reference energies for XPS peak fitting. For the specific case of the experimental data in Figure 1, we can conclude that the peak attributed to sp^3^ motifs in the a-CO_*x*_ sample can be easily explained by the presence of a strongly shifted sp^2^ peak. In fact, as we will discuss in Section 5, none of our attempts to generate computational a-CO_*x*_ samples led to a significant presence of sp^3^ motifs in any of the structures, suggesting that a-CO_*x*_ is probably mostly sp^3^-free. We note in passing that, by virtue of the same argument, any possible sp^3^ peak would anyway also be significantly shifted toward higher energies with respect to the reference value. Values of this shifting will be shown in Section 5.3.

### 2.2 Removing the Ambiguity in Deconvolution of Spectra

XPS analysis is rife with deconvolution schemes, all giving different peak proportions, muddying the deduction of material structure.^37−39^ XPS analysis of carbon chars from Smith et al.^42^ shows the pronounced effect of these deconvolution schemes on the interpretation, predicting different amounts of carbon and oxygen functional groups based on the deconvolution protocol used. Furthermore, there is a lack of self-consistency in many such approaches: the amount of oxygen surmised from the deconvolution of the C 1s spectra is not always consistent with the actual amount of oxygen in the sample. The lack of a universal scheme comes from multiple factors. Of note is that, for deconvolution, an assumption is made: similar environments only contribute to the CEBEs within a narrow range of the reference value. Given a motif structure in the material which is similar to a reference (e.g., sp^2^ carbon), which is now influenced by other atomic species (e.g., environmental oxygen) or defects in the local environment, there are *shifts* to the CEBEs which *cannot* be accounted for by experimental deconvolution techniques, as the reference is fixed. This is precisely the situation we exemplified in the previous section with experimental a-CO_*x*_ data from the literature and our computational GO experiment.

Combining DFT and experimental data, one can give estimates of the CEBE shifts for the *molecular* references used in the deconvolution,^42^ however the issue remains: these shifted molecular references will not have the same CEBEs as similar environments in the bulk material.^21^ This is further complicated with regard to the interpretation of amorphous materials, for which the references will have even less similarity with the true environments present in the material. Thus, on the one hand, a truly *universal* library based on just a few reference motifs cannot be made; on the other, traditional peak assignment leads to highly arbitrary fits whenever too many reference peaks are used^38^ (as famously put by John von Neumann,^43^ “*with four parameters I can fit an elephant, and with five I can make him wiggle his trunk*”).

To obtain an unambiguous deconvolution of an XPS spectrum and to avoid the numerous pitfalls listed above, one can opt for another approach: given a hypothetical atomic structure which matches the experimental sample, one could use a computational model, ideally cheap to evaluate, to predict the CEBEs as a function of the local atomic environment, from which one can readily obtain the XPS spectrum. This spectrum is essentially a high-resolution histogram of CEBEs from a given atomic structure. The benefits of this are multifold: (1) we know exactly each local environment contribution to the XPS spectra; (2) each predicted CEBE has the local environment shifts accounted for; (3) analysis of environment similarity in the structure allows for an *unambiguous* deconvolution of the XPS spectrum into motif contributions. In our previous work,^24^ we showed that an ML CEBE model, built on a careful combination of DFT and *GW* data, achieves the prescribed requirements for quantitative accuracy and computational efficiency outlined above. However, such a model still requires that the user provides an input atomic structure. Therefore, the “only” problem that remains to be solved is how can one find a *realistic* atomic structure whose predicted XPS spectrum matches the experimental one? Solving this problem requires a structure-generation protocol that produces structural models which are *simultaneously* low in energy and reproduce the correct (experimental) XPS spectrum.

## 3 Matching Atomic Structure to Experimental Data

The problem of matching a structure to experimental data is within the realm of reverse MC (RMC) techniques, first developed by McGreevy and Pusztai,^44^ to find the structure of liquid argon based on an experimental pair-correlation function. These methods typically match structure to experimental data only by moving atoms and evaluating a Metropolis acceptance criterion. This criterion is based solely on a measure of the agreement with experiment for the given observable, thus without regard to whether the structure is physical, i.e., low in energy for the given thermodynamic conditions. To circumvent this—without having to evaluate the total energy—the addition of constraints is necessary. These range from user-defined, material-specific, bonding constraints^45^ to adding multiple sets of experimental data.^46^ However, these models are typically under-constrained^47^ and can give unphysical results. A natural way to determine the soundness of configurations during simulation is to use the energy from an interatomic potential. This ensures the system remains in sensible regions of configuration space, while matching experimental data. This strategy is followed in hybrid RMC (HRMC) approaches,^48^ which have thus far only been done by combining empirical potentials and experimental observables, with the limitation that both must be amenable to cheap computation with simple analytical expressions [e.g., pair-correlation functions and X-ray diffraction (XRD)].^47−51^ A recent example of full HRMC is its application to amorphous calcium carbonate,^52^ where the effective interactions could be well described with a relatively simple force field and the inverted experimental data was based on XRD analysis. An alternative way to incorporate experimental diffraction data to structural optimization, while retaining DFT accuracy for the description of the PES, is to combine RMC and DFT heuristically, thus reducing the number of ab initio steps that need to be carried out, as done for a-Si and GeSeAg in ref (53).

The problems with empirical potentials have been widely stated and will not be commented on here in detail. This includes detailed comparisons of their performance against the Gaussian approximation potential^3^ (GAP) framework used here, even for the case of simulating disordered carbon materials specifically.^54−56^ In brief, these potentials cannot accurately reproduce the PES except for in those regions of configuration space for which they were optimized. They are usually parametrized for specific materials and can fail spectacularly, or even catastrophically (“blow up”, in jargon), when configurations are out of the scope of the fit. This is exacerbated when performing MC simulations, which proceed by evaluating sequential energy differences. Hence, there will be large errors in the acceptance criterion and consequently the accepted configurations, which can lead to unrealistic structures. MLPs, on the other hand, can reproduce the ab initio PES accurately, given the inclusion of enough suitably chosen training configurations. A sufficiently general potential would overcome the need for complex, user-defined, material-specific constraints as the potential “knows” what configurations are unphysical, which is reflected in the total energy. We note in passing that poorly designed MLPs are also prone to blowing up, and that this is actually more common in MLPs than in empirical potentials because of the significantly more complex functional form of the potential.

We propose an alternative way to navigate configuration space in order to generate structures which are consistent with experimental XPS data *and* low in energy with regard to ab initio calculations, allowing for reliable and accurate XPS deconvolution and experimental structure determination. We use a machine-learned XPS model—which is informed by *GW* theory—and a CO GAP to predict experimental structures of a-CO_*x*_. XPS spectra predictions are matched to experimental XPS spectra by generalizing the HRMC approach to encompass GCMC simulations, using energies from the CO GAP and XPS spectral dissimilarities as inputs. This synthesis of ML models and experimental data allows for structure prediction at far larger scales than DFT, all while having *ab initio*-level accuracy. Furthermore, this method allows one to dispense with inaccurate deconvolution schemes which plague experimental XPS analysis, providing deeper structural insight into XPS experiments.

## 4 Materials and Methods

### 4.1 ML Potential

To model the PES of the carbon–oxygen system, we rely on a GAP trained from data computed using DFT with the Perdew–Burke–Ernzerhof^57^ (PBE) exchange–correlation functional (PBE–DFT). All the DFT calculations are done with the VASP code,^58,59^ with all the technical parameters given in the Supporting Information. The details of the GAP theoretical and methodological framework can be retrieved from the literature.^4,60^ Briefly, GAP uses GPR/KRR to learn and then predict the potential energy landscape of a system of interacting atoms as a function of atomic descriptors, usually two-body (2b), three-body (3b) and many-body (mb; formally equivalent to an ensemble of 3b descriptors).^61^ Our GAP architecture for the CO potential incorporates:

1 2b descriptors with 4.5 Å cutoff for the C–C, C–O and O–O interactions;
2 3b descriptors with 2 Å cutoff for the six possible permutations of C and O triplets, where a central atom is singled out and the descriptor is invariant with respect to permutations of same-species atoms for the other two (CCC, CCO, COO, OCC, OCO, and OOO, with the first symbol indicating the central atom);
3 mb soap_turbo descriptors,^62^ a modification of the smooth overlap of atomic positions^63^ (SOAP) descriptor, with 4.5 Å cutoff. One SOAP descriptor is added for each species, with both species visible—i.e., the carbon-centered SOAP descriptor is sensitive to both C and O neighbors, and so is the oxygen-centered SOAP descriptor;
4 Tabulated “core” potentials for the C–C, C–O and O–O interactions, explicitly describing the highly repulsive regime when two atoms are down to 0.1 Å from each other. The explicit inclusion of this term improves the stability and accuracy of the GAP fit significantly;
5 Optionally, tabulated long-range pair potentials to describe, in a limited way, van der Waals (vdW) (or “dispersion”) interactions. Currently, long-range C–C, C–O, and O–O dispersion energies use a fixed parametrization. This functionality will be extended to Hirshfeld volume prediction so that environment-dependent vdW correction schemes can be used, as discussed in ref (13).

We refer to this MLP as the CO-GAP. The CO-GAP furnishes us with *local energy* predictions for each atom *i* which are defined as
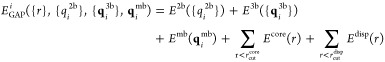
where {*r*} is a set of atomic pair distances with respect to atom *i*, *E*^core^(*r*) and *E*^disp^(*r*) are energies from the core potential and dispersion interactions, respectively, {*q*_*i*_^2b^} and {**q**_*i*_^3b^} are sets of 2b and 3b descriptors pertaining to each pair or triplet of species, respectively, and **q**_*i*_^mb^ is a soap_turbo descriptor. The total energy of a configuration is simply given by the sum of local energies
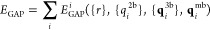


This CO-GAP is freely available from the Zenodo repository.^64^ Future (improved) versions of the CO-GAP will also be added to the same repository, which will retain the whole version history to ensure reproducibility of published results. The CO-GAP can be used to predict energy and forces with TurboGAP,^65^ QUIP,^66,67^ and LAMMPS^68,69^ via its QUIP interface. The modified dynamics with on-the-fly XPS prediction and inclusion of experimental observables discussed in Sections 4.4 and 4.5 is only currently available in TurboGAP.

### 4.2 Iterative Database Generation

The initial training database is constructed by using the a-C database of Deringer and Csányi,^70^ recomputed at the PBE–DFT level. We extend it by adding all possible C_60_ isomers,^71^ the dissociation curves of CO and O_2_, and iteratively generated a-C:O structures of different sizes and compositions. The iterative training procedure is as follows. Initially, before the GAP has “seen” any O-containing carbon structures (beyond the CO dissociation curve), we run three independent ab initio MD trajectories with different mass densities. Initial 3 × 3 × 3 simple cubic structures, containing 24 C atoms and 3 O atoms randomly substituted in the lattice, are quickly quenched from 4000 K down to 300 K over 1 ps. This provides a computationally affordable glimpse at the configuration space spanning from l-C:O down to a-C:O. Ten snapshots from each run are chosen to be included in the training database. A first version of the CO-GAP is fitted from this database, and used instead of DFT to cheaply generate more training data as in the first iteration, but this time sampling 9 different starting configurations varying from 8 up to 64 atoms at a similar composition (∼10% O content). These structures are quenched from 5000 K down to 300 K over a significantly longer 20 ps simulation, collecting snapshots every 2 ps, to avoid highly correlated configurations. These single-point snapshots are used to run PBE–DFT calculations and added to the growing training database. The same procedure is repeated several times, while slowly increasing the maximum O content, until the GAP generates low-temperature structures of arbitrary composition whose predicted energies are close enough to the DFT values.

This first iteratively trained GAP is used in a series of quasi-production runs, with 210 512-atom simple-cubic structures at different densities (1.5 to 2.5 g cm^–3^) and compositions (2.5 to 60% O contents), to generate a-CO_*x*_ structures using a slower quench process^72^ from 3500 K down to 300 K over 100 ps. The final configurations range in structure from a-C:O to a-CO_*x*_ and even “burnt” systems, with lots of CO and CO_2_ molecules spontaneously forming at very-high O content. They are recomputed with PBE–DFT and also added to the training database. The resulting GAP still undergoes a few rounds of iterative training for high-O-containing structures with a CO/CO_2_ removal step at the end (before the DFT calculation). Finally, another few rounds of iterative training are carried out to include “adsorption” configurations, i.e., a-CO_*x*_ structures derived by adding O atoms to a pre-existing graphitic a-C structure on the bridge or top positions, corresponding to the classical ether/epoxide and ketone organic chemistry groups, respectively. In these adsorption iterative rounds, one O atom is placed at the adsorption site, followed by a relaxation and removal of any possible CO and CO_2_ formed molecules, before placing the next O atom and repeating the procedure.

**Figure 3:**
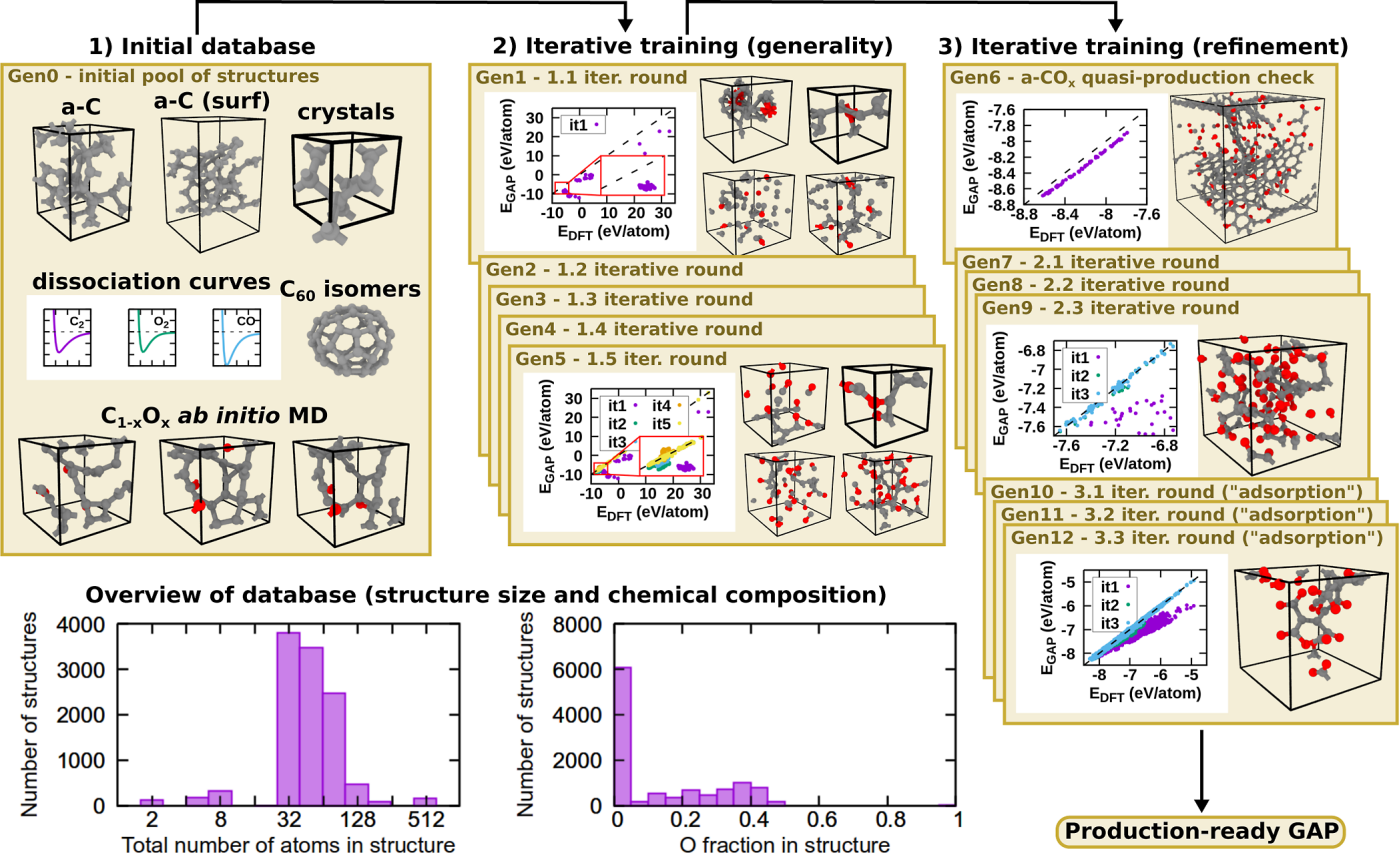
Iterative training workflow followed while developing the CO-GAP. The initial database, which is added for stability and generality, is incrementally improved and refined by adding more purpose-specific and high-O-content structures. The two panels in the bottom-left corner show an overview of the composition of the database, in terms of the size (number of atoms) of the periodic simulation boxes and their stoichiometry.

One of the advantages of this iterative training procedure is that the DFT calculations used to train the next generation of the MLP can be used to compute an unbiased error estimate for the previous generation of the MLP. The whole workflow to train the CO-GAP is depicted in Figure 3, including example structures and scatter plots for selected GAP versions, where GAP predictions are tested for the structures to be added to the *next* generation of the training database. Overall, our training database was incrementally enhanced from generation zero (“Gen0”) until generation 12 (“Gen12”). Going from one generation to the next involves training a new GAP. Thus, the last “production-ready” GAP, trained from the Gen12 training database, is the 13th GAP overall that was trained as part of the iterative process. The histograms on the bottom-left corner of the figure show the overall composition of the *final* training database, in terms of total number of atoms per structure and structure stoichiometry. We note that most of the training configurations have between 32 and 128 atoms, with some very small (2, 4, and 8 atoms) and very large (512 atoms) structures. We also note that a large fraction of structures contain only carbon atoms, taken from the initial training database. This is required because of the large diversity of carbon structures that can be realistically found in nature (in particular in a-C). The relevant size of configuration space for O-containing structures is comparatively small, because there are not so many ways in which O atoms can bind to C atoms to form stable arrangements.

All the fits were carried out with the gap_fit program,^73,74^ part of the QUIP software package.^66,67^ Atomic structure generation, manipulation, MD simulations, etc., were done with the Atomic Simulation Environment^75^ (ASE), different in-house codes, and the TurboGAP program.^65^

### 4.3 Computational X-Ray Spectroscopy

To predict XPS spectra from a-CO_*x*_, we use the SOAP-based XPS model that we have previously developed,^24^ retrained with gap_fit’s “local property” fitting functionality.^73^ This model predicts CEBEs based on the local atomic environment of an atom *i*, which is characterized by a SOAP descriptor **q**_*i*_
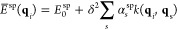
1where  is the CEBE prediction, δ^2^ provides the energy scale, {α_*s*_} is the set of fitting coefficients, and *E*_0_ is an offset energy, ideally chosen close to the training set average. The sp superscript (for “species”) indicates that different models are trained for different species, in our case we can train one model for C 1s CEBEs and another one for O 1s CEBEs; while this paper focuses on the former, the model architecture does not change, and our previous paper discusses both.^24^ In eq 1, {**q**_*s*_} is the set of SOAP descriptors in the sparse set, *s* ∈ {1, ..., *N*_s_}, where *N*_s_ is the number of sparse configurations (see ref (4) for a discussion of sparse GPR in the context of atomistic ML). *k*(**q**_*i*_, **q**_*s*_) is the kernel function, which gives the similarity between the atomic environments of atoms *i* and *s*, ranging between 0 and 1 and given by
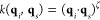
2where ζ = 2 in our case. In the following, we use  for shorthand notation.

The training set was composed of CEBEs which were calculated, using a combination of DFT and *GW* reference data, from bulk material as well as surfaces. This model accounts for the fact that, in solid-state samples, the experimental reference energy is given by the Fermi level of the material (as opposed to the vacuum level used for molecular samples). The training database consisted of a-C, a-C:O, functionalized a-C, a-CO_*x*_, graphene, and reduced graphene oxide (rGO). For further details, see ref (24).

To account for thermal and instrumental broadening, the predicted CEBEs were broadened by a Gaussian of width σ = 0.4 eV, and the resulting data was normalized over the domain of prediction, giving the final XPS spectrum from the contributions of all atoms contained in a computational structural model *S*
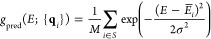
3where *E* is an arbitrary energy along the domain of the spectrum and *M* is a normalization factor to make . While the experimental peak fitting procedure uses peak widths as a way to account for instrumental broadening, thermal broadening *and* structural disorder, our procedure incorporates disorder directly by generating the distribution of atomic environments explicitly. Therefore, our smearing parameter should only account for instrumental and thermal broadening, which should be fixed for given experimental conditions: i.e., given an experimental apparatus and temperature, the induced broadening that we should consider to mimic the convolution of both effects is fixed. Our methodology implicitly incorporates the broadening in the XPS spectrum necessary to account for disorder by explicitly generating the necessary distribution of structural motifs. Thus, within our method, the σ value is chosen based on the broadening that can be found in typical experimental setups, where synchrotron-based XPS spectra will have better resolution that lab-based spectra (see ref (22) for a discussion of σ in the context of comparing simulation to experiment). We expect that the effect of too small/large σ values would be for the structure-generation protocol to counteract it by inducing a broadening/narrowing of the spectral features by spuriously exacerbating the degree of atomic motif disorder/order in the structure. For these reasons, σ is *not* an optimizable free parameter of the model but must be chosen on physically motivated grounds. We further discuss the choice of σ and show the effect of varying this parameter in the Supporting Information.

### 4.4 Combining Experiment and Simulation for Realistic Structure Determination

We can compare the normalized XPS spectrum *g*_pred_(*E*; {**q**_*i*_}) to the normalized experimental spectrum *g*_exp_(*E*), since *absolute* XPS intensities are arbitrary. We can define a “dissimilarity” metric, , that varies from 0 to 1, which we take to be a sum of squared differences
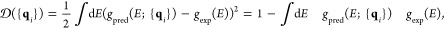
4where the second equality is valid because of the normalization of the spectra.

Defining an energy scale as γ, we can create the pseudoenergy , which allows for construction of the modified total energy of the system
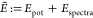
5where *E*_pot_ ≡ *E*_GAP_ (in our case) is the potential energy of the system. Although the focus in this paper is on XPS, we could more generally define the modified energy from an arbitrary linear combination of contributions, each based on an experimental observable, as .

*E*_spectra_ acts as a penalty term, increasing the energy with increasing spectral dissimilarity. From a RMC perspective, where the focus is on matching experimental data regardless of atomic total energies, the *E*_pot_ term acts as an energy-based constraint on the atomic positions: high-energy atomic configurations are penalized. By optimizing Ẽ with an appropriate γ, we perform a multiobjective optimization with respect to the atomic positions, favoring the generation of atomic configurations which *simultaneously* match the experimental XPS spectrum and are low in energy. An additional benefit of this multiobjective optimization is that the CEBE model only needs to be quantitatively accurate for low-energy structures, as extrapolation errors for high-energy structures do not impact the optimization because the potential energy constraint discourages these structures from being accepted. This is illustrated in Figure S5 of the Supporting Information.

For computational efficiency, we use the same structural mb descriptors {**q**_*i*_} for the CEBE model as we do for the CO-GAP. This speeds up the simulation: the *E*_pot_ term is calculated first, which requires a descriptor calculation for each atom in the structure. This step has the highest computational cost incurred during simulation. As the relevant descriptors have already been calculated, the only necessary calculation for prediction of the XPS spectra is the evaluation of the kernel function between these descriptors and those of the sparse set corresponding to the CEBEs, eq 1.

One of the purposes of XPS analyses in multispecies materials is to determine the elemental composition of the samples. Therefore, being able to reliably tune the relative number of atoms of different species in our simulations is essential. For instance, the XPS analyses of a-CO_*x*_ by Santini et al.^31^ show that the O/C ratio varies significantly with O_2_ partial pressure. For comparison, we must generate representative structures at a range of oxygen concentrations. A natural way to generate these structures is to start from pure a-C and perform GCMC simulations with different chemical potentials with our modified energy *Ẽ*, as discussed next.

### 4.5 Modified GCMC

In a GCMC simulation, a system of interest is at fixed volume *V*, allowed to thermalize by contact with a heat bath at temperature *T* and exchange particles with an infinite reservoir at a chemical potential μ, forming a constant (μ, *V*, *T*) ensemble. We perform GCMC using a Markov chain: starting from an initial pure a-C structure, we generate trial configurations by either randomly displacing a particular atom, or inserting/removing oxygen into/from a random position, respectively. These trial configurations are either accepted or rejected using the standard acceptance criteria^76^ for particle displacement/insertion/removal
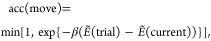
6
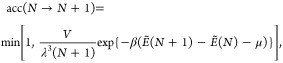
7
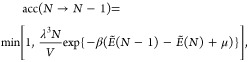
8where λ is the thermal de-Broglie wavelength, given by , and β = 1/*k*_B_*T*. A trial configuration is accepted if the corresponding acceptance criterion is greater than a random number *r* ∈ [0, 1]. We then repeat the procedure with the last accepted configuration until the maximum number of iterations has been reached.

Thus, the steps for performing GCMC simulations to optimize the XPS spectrum are as follows:

1 Generate trial configuration from a randomly chosen MC step type (insertion, removal, move);
2 Evaluate *Ẽ*, from eq 5;
3 Evaluate the corresponding acceptance criterion, one of eqs 6, 7, and 8, and compare to *r*;
4 Repeat until the target number of MC iterations has been reached.

### 4.6 Deconvolution of a-CO_*x*_ Spectra

We deconvolved C 1s XPS spectra by considering the resultant structures of GCMC simulations as undirected graphs: atoms were nodes differentiated by species, and bonds were edges. This was achieved with the NetworkX package in Python^77^ in conjunction with ASE.^75^ Bonds were defined by overlaps of atomic spheres, where an atomic radius of 0.912 Å was used for C and a radius of 0.792 Å for O—this was done using the in-built ASE functions NeighborList and natural_cutoffs functions with mult = 1.2. For each carbon, a graph was made which contains its first nearest neighbors and, if an oxygen was found, the neighbors of the oxygen. A reference graph database was created from the connectivity of standard motifs (e.g., sp, sp^2^, ether, etc.). Subgraph isomorphisms were sought between the reference graphs and each carbon subgraph in the structure in a hierarchical fashion, from the most complex to the least (in the order: carbonate, peroxide, ester, epoxide, ether, ketone, CO_2_, CO, sp^3^, sp^2^, and sp), with the most complex subgraph isomorphism being chosen. This was sufficient to associate each carbon with a motif. C 1s CEBEs were grouped according to these motifs, allowing for the delineation of each motif contribution to the XPS spectrum.

## 5 Structural Model Generation

In this section, we explore different approaches to generating a-CO_*x*_ and assess their ability to yield results in agreement with experiment. In particular, the challenge in a-CO_*x*_ atomistic structure prediction is to generate metastable oxygenated a-C structures with very high O content. Although the most realistic strategy would be to carry out simulated deposition as has been done for a-C in the past,^55,72,78,79^ trying to mimic the experimental growth process, this is also the most sophisticated and time-consuming method, in terms of both human and computer time required. Therefore, we explore alternative, cost-effective strategies and compare them to the modified GCMC approach developed here.

### 5.1 Melt-Quench Generation

For a-C, melt-quench MD simulations are arguably the most straightforward type of atomistic structure generation procedure, after a simple geometry optimization.^72,78,80−82^ Melt-quench simulations of a-C start out from a high-temperature liquid sample (“melt”) and rapidly cool it down (“quench”) to trap the atoms into metastable configurations. There is an intermediate high-temperature annealing step, where the system is allowed to equilibrate and find more energetically favorable configurations. Depending on the duration of the annealing step and the quenching rate, disordered carbons with different degrees of graphitization are obtained.^54,70,72,83−85^ In addition to the choice of a temperature profile with an MD thermostat, whether the density of the system is fixed or an external pressure is applied via an MD barostat will also affect the properties of the resulting material.

**Figure 4:**
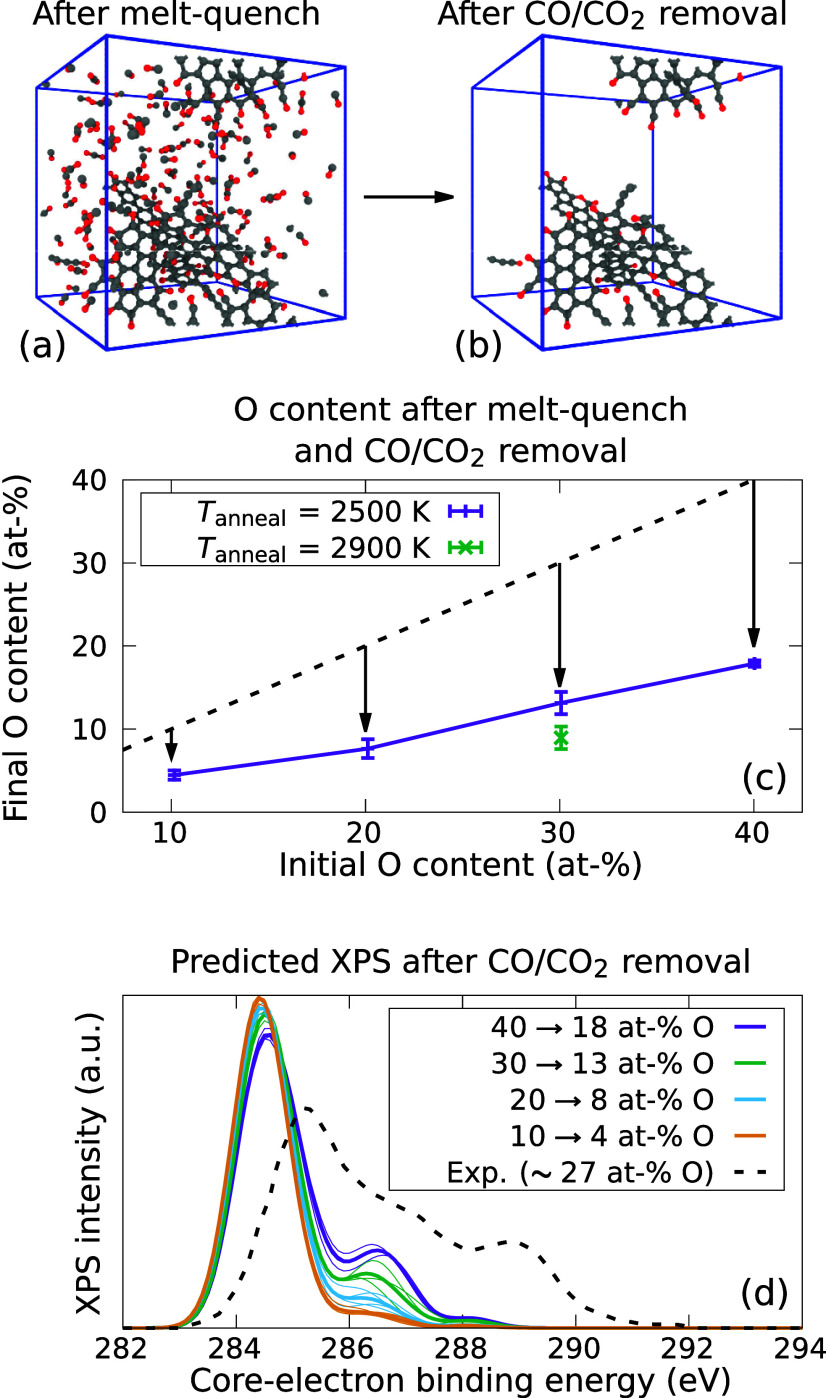
Example CO melt-quench sample after (a) high-temperature annealing and quenching and (b) subsequent removal of molecular species. (c) Final O content in the samples after CO/CO_2_ removal as a function of the original O content. (d) Predicted XPS corresponding to the melt-quench samples after molecular species removal, compared to the target experimental spectrum, where we have indicated the O content inferred with our modified GCMC approach in Section 5.2.

Given the past success and popularity of melt-quench simulations for a-C structure generation, a naive attempt at generating computational a-C:O and a-CO_*x*_ samples using this method appears as an obvious choice. Therefore, we carried out melt-quench simulations of a-C:O formation with our CO-GAP. We start with randomly initialized systems with fixed C/O ratios (10, 20, 30, and 40 at-% O contents) and apply the following temperature profile, while applying a 1 bar barostat: 10 ps of liquid thermalization at 5000 K, 100 ps of high-temperature annealing at 2500 K, and 10 ps of low-temperature annealing at 300 K. At each C/O ratio, 3–4 samples are simulated to assess the effect of random initialization on the results. Invariably, the structures that we obtained consisted of graphitic flakes with O passivation along the edges and a large degree of CO and CO_2_ formation. An example structure is shown in Figure 4 before (a) and after (b) the CO and CO_2_ molecules were removed. The trends show that most of the O atoms originally present in the simulation box are being used to generate the thermodynamically more stable molecules, rather than being trapped into the metastable amorphous motifs (Figure 4c). We repeated a series of simulations at a higher annealing temperature (2900 K), clearly showing an even more pronounced formation of molecular species.

As expected, the samples yield, after removing the molecular species, XPS signatures that are very different from Santini’s experimental sample which we aim to reproduce (Figure 4d), for which we estimate in Section 5.2, with our modified GCMC simulations, an O content ∼27%. Thus, melt-quench simulations of CO mixtures seem to favor the formation of the thermodynamically stable molecular products and produce low-O-content a-C:O structural models, below ∼20 at-% O. The inability of high-temperature annealing protocols to produce experiment-compliant structures draws parallels with how simulations of undoped tetrahedral a-C systematically failed to generate high-sp^3^ samples matching experiment until high-accuracy explicit deposition simulations became feasible with MLPs.^55,72,79^ In the case of a-C:O and a-CO_*x*_, melt-quench protocols further exacerbate this issue because the favoring of high-entropy thermodynamically stable products in the C–O system leads to molecular species formation, far away from the target solid-state samples, whereas for pure carbon it simply favors solid-state samples with higher graphitic content (i.e., lower sp^3^/sp^2^ ratios than in experiment), as the thermodynamically stable product is graphite. Therefore, melt-quench simulations are not a good surrogate for the (nonequilibrium) deposition mechanism that takes place during the experimental a-CO_*x*_ synthesis. Hence, we developed the modified GCMC scheme, which is detailed next.

### 5.2 Modified and Unmodified GCMC

#### 5.2.1 Choice of Initial Structures

**Figure 5:**
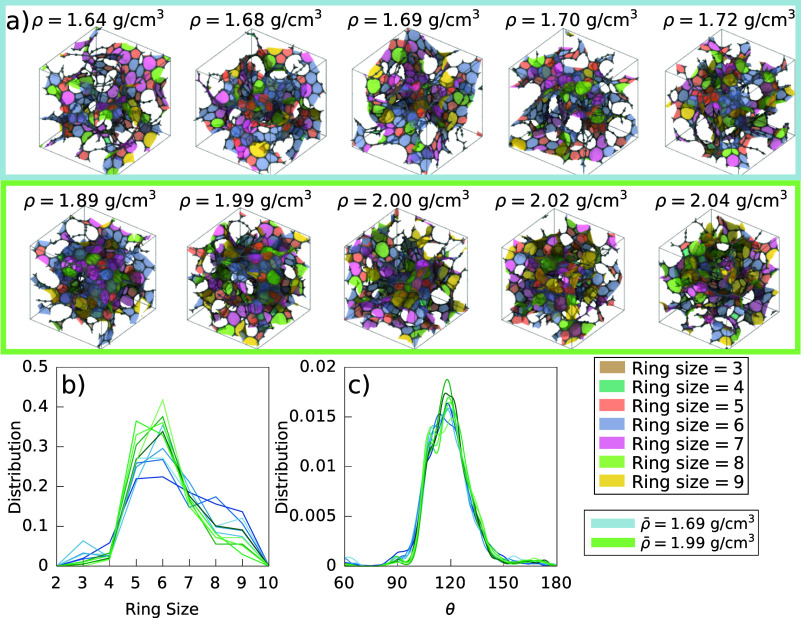
Two groups of initial structures used in the simulations, which have distinct densities. (a) Structures with surfaces colored according to the number of members in the carbon ring. The ring search was capped at 9-membered rings. (b) Ring-size distributions. (c) Bond-angle distributions. Saturation of color enumerates the structures in the group, the lightest being first and the darkest being last.

Ten 512-atom a-C structures were used for the initial configurations of the modified GCMC simulations, as seen in Figure 5 a. These were generated from a melt-quench procedure, similar to ref (85). The structures can be grouped into two distinct average densities, ρ̅ ≈ 1.69 g cm^–3^ and ρ̅ ≈ 1.99 g cm^–3^. The structures of lower density have a slightly more diverse ring structure, as seen in Figure 5b. As such, their bond-angle distribution differs slightly from those of higher density, in Figure 5c.

Ten separate MC XPS optimizations were run for 500 000 steps for each structure at a given chemical potential μ ∈ {−10, −5.16, −3, 0} eV, with a temperature of 300 K. The chemical potential of −5.16 eV can be related to a reference of half the DFT binding energy of diatomic oxygen at 1 atm of pressure at 300 K and is taken from Samanta et al.^86^ Two C 1s XPS spectra of a-CO_*x*_ were used for fitting, both taken from Santini et al.:^31^ for the chemical potentials of μ ∈ {−10, −5.16} eV, the XPS spectrum of low oxygen content—O/C ratio ≈ 0.1, according to ref (31)—was used with γ = *N*_C_γ_C_^low–O^ where γ_C_^low–O^ = 39.0 eV per carbon atom and *N*_C_ was the number of carbon atoms; for the chemical potentials of μ ∈ {−3, 0} eV, the XPS spectrum of highly oxygenated a-CO_*x*_—O/C ratio ≈ 0.6, according to ref (31)—was used with γ = *N*_C_γ_C_^high–O^ with γ_C_^high–O^ = 19.5 eV per carbon atom. Unoptimized XPS simulations were performed on exactly the same structures with γ = 0 eV.

#### 5.2.2 Convergence of GCMC Runs

**Figure 6:**
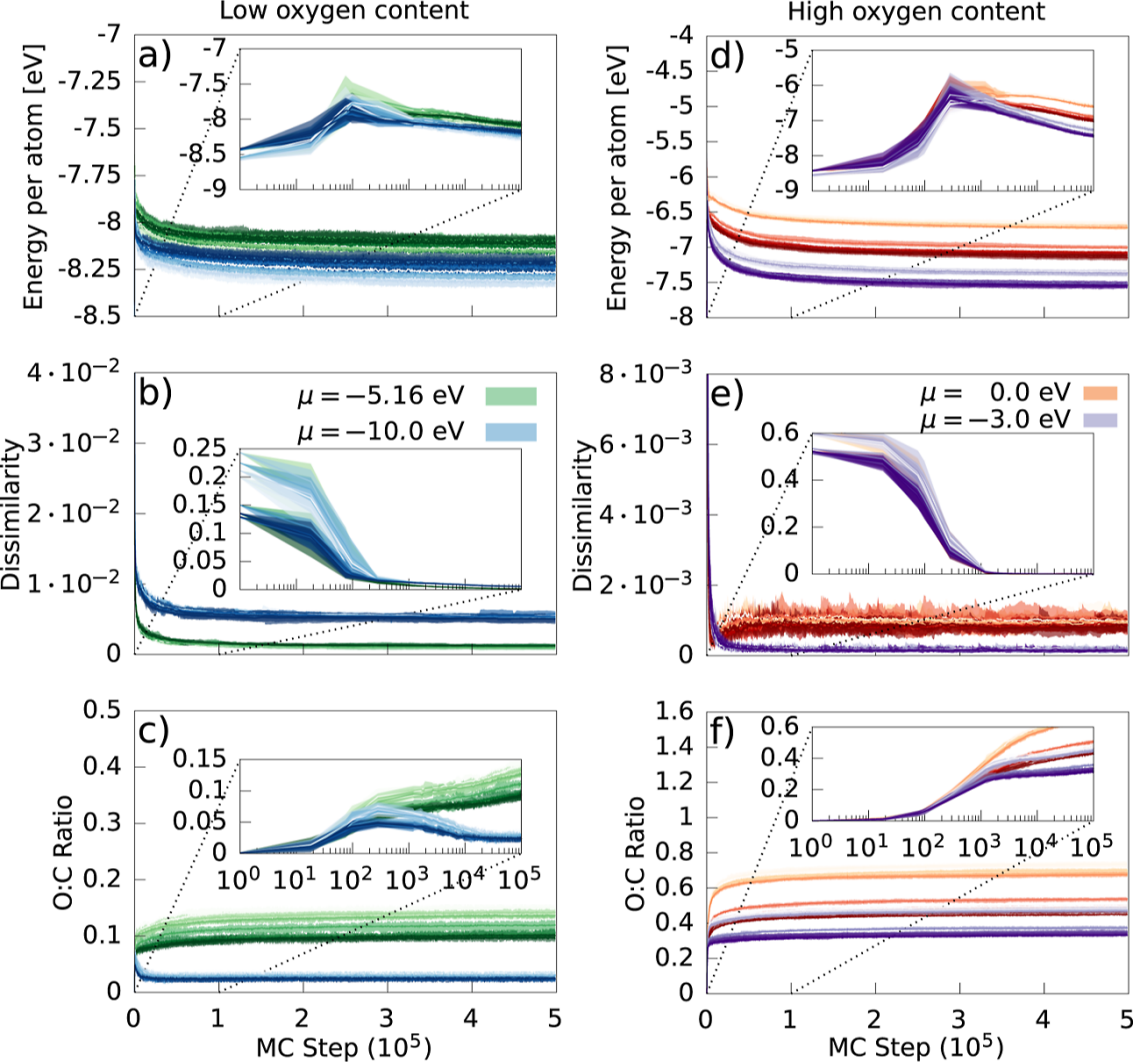
Modified GCMC runs for different chemical potentials to generate a-CO_*x*_ structures, with XPS optimization for the low- (left-hand-side panels) and high-oxygen-content (right-hand-side panels) XPS spectra. Each line represents a different structure. Solid lines are the averages over the runs at that particular time step, with shaded regions denoting the extrema of the data. (a/d) Energy per atom. (b/e) Dissimilarity. (c/f) O/C ratio. Inset are logscale plots, showing the initial equilibration of the system.

Figure 6 shows the evolution of the local energy per atom, the dissimilarity, and the O/C ratio during the modified GCMC runs. The left-hand-side panels show structures optimized with respect to the low-oxygen-content a-CO_*x*_ XPS spectrum, seen in Figure 1a, and the right-hand-side panels with respect to the high-oxygen-content XPS spectrum, seen in Figure 1b. The color saturation of the lines reflects the density of the structures, which are in the order of left-to-right, top-to-bottom in Figure 5: lighter lines correspond to lower density structures, and darker lines to higher densities. Convergence in the energy per atom, dissimilarity, and O/C ratio was reached by 2 × 10^5^ steps for all simulations.

Generalized Hamiltonian MC initially acted to match the spectra by increasing oxygen content, after which there was more structural relaxation and equilibration. The energy per atom increased with oxygen content as the spectral dissimilarity decreased up to 10^3^ MC steps. After this point, the deviations of the spectra were small and the local energy decreased. We show in Figure S6 of the Supporting Information that the change in the potential dominates that of the change in*E*_spectra_ for configurations with a sufficiently low spectral dissimilarity. The change in potential is generally positive for resultant structures, demonstrating atomic relaxation.

There was a diversity of O/C ratios found at a given chemical potential. The aptitude for oxygen insertion was highly dependent on the density of the structure, with those of lower density preferring a greater amount of oxygen. This is apparent in the bifurcations of the O/C ratio in Figure 6c. Structures with greater amounts of oxygen had a higher energy per atom overall.

The original analysis of the low-oxygen-content a-C sample found in Santini et al. gave an O/C ratio of 0.1, which agrees with our low-density a-CO_*x*_ simulations of μ = −5.16 eV. The a-CO_*x*_ results of Santini et al. gave O/C ratios of 0.4–0.8, which are comparable to all our μ = 0 eV structures and the low-density structures found at μ = −3 eV. Below we show that, although it is possible to generate metastable a-CO_*x*_ structures computationally with similarly high O/C ratios without XPS optimization, the resultant XPS spectra are very far from experiment and the actual O/C ratio which matches the experimental spectrum is lower.

#### 5.2.3 Modified vs Unmodified GCMC Results

**Figure 7:**
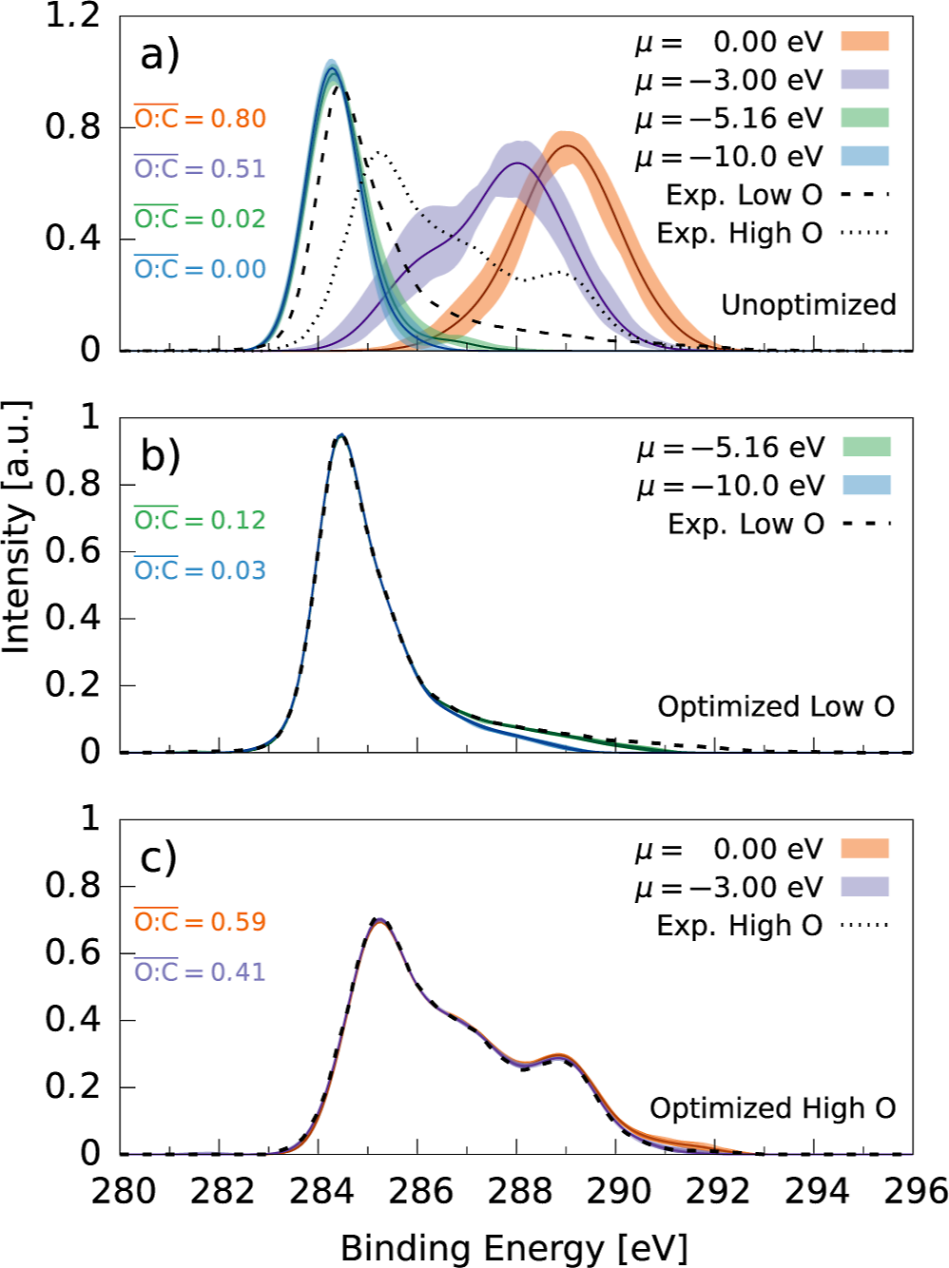
C 1s XPS spectra of a-CO_*x*_ structures (a) without and (b/c) with XPS optimization. Experimental data taken from ref (31) (b,c) correspond to optimization of the low- and high-oxygen-content spectra, respectively. Filled curves denote the maximal extents of the individual runs at the given chemical potential; lines give the mean value of the spectra.

Generalized Hamiltonian MC optimization resulted in almost perfect agreement of the XPS spectra for all structures. The XPS spectra from the unoptimized XPS and optimized XPS runs can be seen in Figure 7. The evolution between a low O-content spectrum and a high O-content spectrum along a single GCMC run is shown in Figure S1 of the SI. The unoptimized, low-oxygen-content XPS spectra had good agreement with experiment. The modal peak position of 284.3 eV is at a slightly lower value than the experimental 284.5 eV, which is expected due to a lack of CEBE shifts from high oxygen content seen in the other samples. XPS optimization resulted in spectral agreement primarily by increasing oxygen content to reproduce the high CEBE tail and causing positive CEBE shifts. Optimization completely inhibited the large deviations from experiment present in the unoptimized high-oxygen-content spectra, seen in Figure 7a. The secondary peak at 289 eV is reproduced well by the optimized spectra, and related to the inclusion of oxygenated species.^31^ The origin and magnitude of CEBE shifts exhibited in the spectra will be explored in Section 5.3.

**Figure 8:**
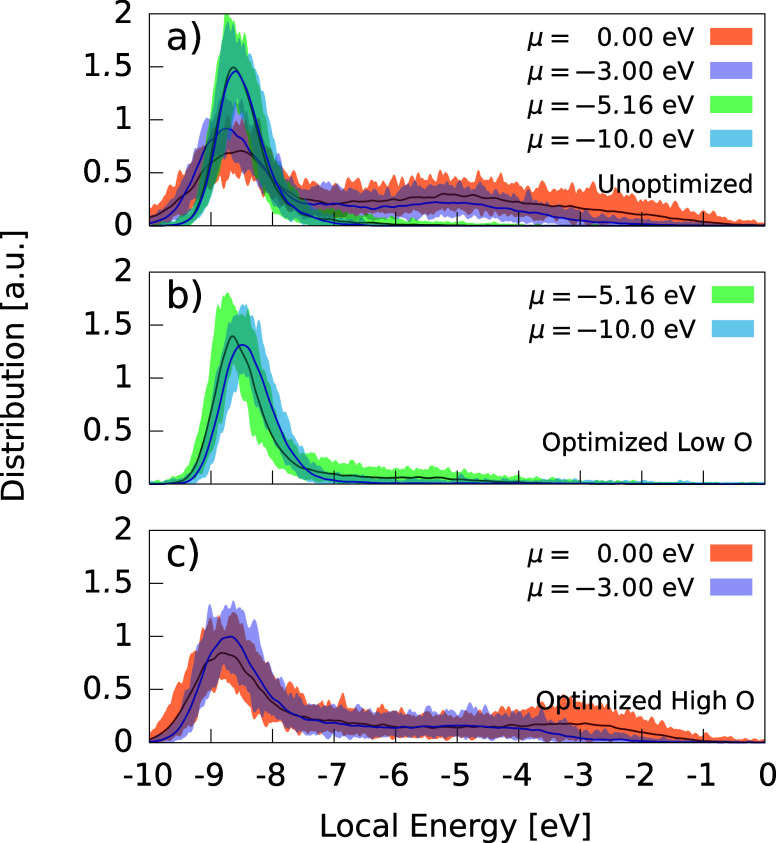
Atomic local-energy distribution of a-CO_*x*_ structures with and without XPS optimization. Filled curves denote the maximal extents of the individual runs at the given chemical potential; lines give the mean value of the distribution. (a) Without XPS optimization. (b) Optimized to low-O a-CO_*x*_ XPS. (c) Optimized to high-O a-CO_*x*_ XPS. These plots are smoothed by Gaussian kernel density estimate.

XPS-optimized structures had low energies, with local-energy distributions (Figure 8b,c) comparable to that of standard GCMC (Figure 8a). The position of the local-energy maxima in unoptimized and XPS-optimized structures was similar, with only slight shifts upward in the latter for μ = −10 eV and μ = −3 eV. This suggests that some carbon environments in these simulations had to be displaced away from lower energy metastable configurations to fit the experimental XPS spectra. The deviations in peak height can be attributed to oxygen content: greater amounts of oxygen caused a more pronounced broadening of the local energy distribution.

From these results, we conclude that the inclusion of the spectral penalty term in eq 4 imposed a sufficient constraint on the MC sampling to promote spectral agreement, while also allowing for the generation of low-energy structures. The action of this constraint caused the total oxygen content to differ from that of the unoptimized XPS simulations, allowing for an adequate reproduction of the XPS features present in the experimental spectra. The total oxygen content increased for the low-oxygen XPS simulations to obtain the high CEBE tails, while oxygen content decreased for the high chemical potential simulations, to inhibit large sp^2^ transformations and sizable oxygen-induced CEBE shifts. For further reference, a comparison between XPS-optimized and -unoptimized structures, at similar oxygen content, is given in the Supporting Information (Figure S2), showing a marked difference.

The experimental growth process for many a-C materials proceeds through physical vapor deposition. This is a highly energetic, nonequilibrium process. The products formed will generally be metastable, rather than thermodynamically favorable. Structure generation methods which proceed by sampling traditional partition functions will tend to produce more thermodynamically favorable structures which maximize the entropy—as demonstrated by the melt-quench protocol in Section 5.1. The generalized Hamiltionan approach provides a means to efficiently search the configuration space of the metastable, experimentally viable structures.

### 5.3 Motifs and Deconvolution

**Figure 9:**
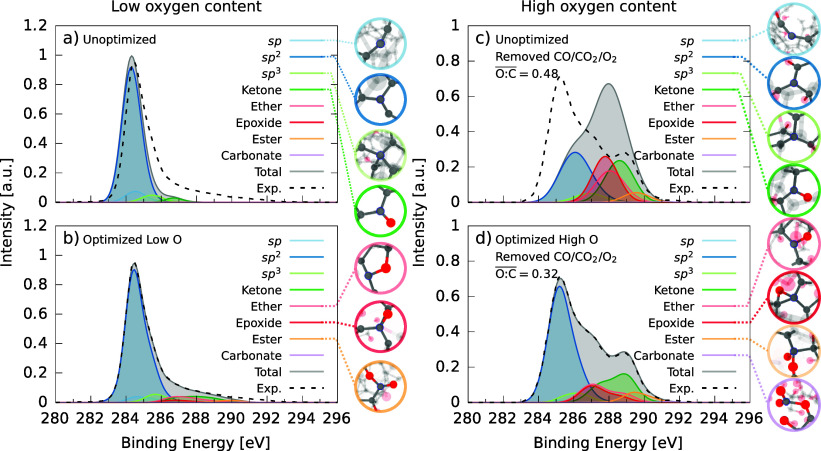
Averaged deconvolution of a-CO_*x*_ structures at low oxygen content with μ = −5.16 eV (left-hand-side panels) and high oxygen content with μ = −3 eV and molecular CO_2_/CO/O_2_ removed (right-hand-side panels). Images of characteristic motifs found in the structures are shown on the right of each panel. (a/c) The unoptimized XPS spectra. (b/d) With XPS optimization. Contributions to the high CEBE range come from the formation of oxygenated motifs.

The deconvolution of the low-oxygen-content spectra, Figure 9a,b, showed that sp^2^ carbon motifs dominate the lower end of the spectra for both unoptimized and optimized simulations. sp^2^ composed the majority of the large peak at ∼285 eV, similar to the experimental deconvolution results in Figure 1a. The proportions of sp, sp^2^, and sp^3^ motifs were similar between optimized and unoptimized simulations. XPS optimization added small amounts of oxygen (in the form of ketone, ether, and epoxide groups) that contributed at higher binding energies than sp^3^ to account for the large tail of the experimental XPS spectrum.

The effect of XPS optimization at high oxygen content was to preserve a large proportion of sp^2^ motifs, see Figure 9c,d. For both the unoptimized Figure 9c and optimized simulations Figure 9d, sp^2^ motifs gave the largest contribution to the XPS and composed the lower CEBE ranges, in addition to sp^3^. Many sp^2^ motifs and all sp motifs present in the initial a-C structures were transformed to ketone, ether, and epoxide groups, which constituted the middle and upper XPS range. These contributions all significantly overlap. Ester and carbonate groups were only found in the high CEBE ranges. XPS optimization inhibited the number of sp^2^ transformations which could take place, resulting in more sp^2^ motifs in comparison to the unoptimized simulations. The oxygenated groups can be attributed to the secondary peak in the experimental XPS spectrum. The CEBE ordering of motif contributions conformed with experimental deconvolution refs (31 and 42).

Molecular species CO, CO_2_, and O_2_ were formed in these simulations. The formation of O_2_ was an artifact of the simulations being performed at a high chemical potential. The removal of these molecules did not noticeably impact the resulting XPS spectrum: compare Figure 7c and Figure 9.

**Figure 10:**
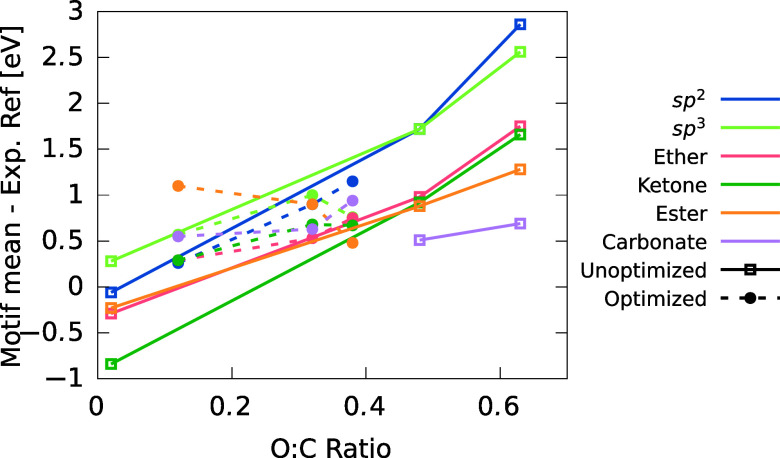
Difference of the mean CEBE of a given motif with experimental references subtracted, taken from refs (36 and 87), as a function of O/C ratio. Experimental references of Moulder et al.^36^ are taken to be the midpoint of the ranges. Unoptimized simulations are the solid lines and optimized the dashed. CEBE shifts are linear with oxygen content. There were no carbonate groups formed at low oxygen content in the unoptimized simulations, hence the lack of data in this range.

CEBEs increased linearly with bonded oxygen content. The mean CEBE motif contributions at different oxygen contents are compared to experimental references in Figure 10. The deviations from experimental references come from at least two factors: (1) The experimental references are molecular: these are bulk simulations; (2) oxygen is electronegative, which reduces the potential locally, therefore increasing the CEBEs with oxygen content.

### 5.4 Discussion

Our XPS optimization method provides a means to efficiently generate experimentally viable a-CO_*x*_ structure candidates for different input experimental XPS spectra. Using a dissimilarity measure of the spectra, an efficient search of viable structures is achieved by a traversal of configuration space that is sensitive to both the total energy of the structure and spectral agreement. It has distinct advantages over using the melt-quench MD procedure: (1) the XPS spectrum of the generated structure matches that of experiment, even after the removal of extraneous molecules; (2) high-oxygen content, metastable structures can be generated: the melt-quench procedure does not allow this due to uncontrolled carbon burning—the generation of CO/CO_2_. The inability of unmodified GCMC or melt-quench procedures to produce structures compatible with experimental spectra is a consequence of the large number of equilibrium atomic configurations which are possible for amorphous structures. The probability of obtaining experimentally feasible metastable structures (i.e., those compatible with the experimental synthesis process) is small without guidance from experimental data.

XPS is limited by the fact that structurally dissimilar motifs can sometimes contribute to the spectral intensity at the same CEBE. This is not a computational artifact, but an intrinsic characteristic of XPS as an analytical method, and it also plagues experimental deconvolution techniques. Thus, multiple structural candidates can match the chosen XPS spectrum, even within the approach introduced here. This shows that a large configuration space is still available after restriction by experimental XPS spectrum conformation, in the case of amorphous materials. This has been demonstrated recently by use of a diffusion model to generate a-C structural candidates which match X-ray absorption near-edge spectroscopy data.^30^ To determine a unique structure, more experimental data can be included to the multiobjective optimization to restrict the search in configuration space, as is done in multiple RMC approaches.^47−51^ The TurboGAP code^65^ already allows for this possibility with regard to XRD and small-angle X-ray scattering data, and it will be investigated in future work.

One is not limited to exploring the experimentally constrained configuration space by MC techniques. We expect that evolutionary algorithms—such as USPEX^88−90^—would allow for sufficient sampling of this space to find experimentally viable, low-energy structures.

## 6 Conclusions

Oxygen can induce significant positive CEBE shifts in amorphous carbon motifs which interfere with experimental XPS interpretation. These shifts arise from C 1s core electrons experiencing a more negative potential due to negatively charged oxygen in the local environment, resulting in higher CEBEs overall. This effect renders structurally similar experimental CEBE references incomparable, leading to an erroneous XPS interpretation upon deconvolution.

The effect of these shifts on experimental interpretation is evident in the differences of the high-oxygen content deconvolution from the experimental analysis of Santini et al.,^31^Figure 1b, and that of this work, Figure 9d. Santini et al. suggested that a transformation of sp^2^ to sp^3^ carbon takes place in a-CO_*x*_ upon a 5 min anneal with temperature increasing from 100–500 °C. The sp^2^ reference energy was not shifted to account for the presence of oxygen, leading the authors to deduce that all nonoxygenated carbon was of sp^3^ type from the XPS spectrum deconvolution. Significant numbers of sp^2^ → sp^3^ transitions did not occur in our simulations, and we showed that the effect of environmental oxygen is sufficient to explain the presence of a strongly shifted sp^2^ peak in a-CO_*x*_ without the need to invoke the presence of sp^3^ carbon.

We expect that similar shifts of CEBEs occur in materials with other electronegative species, with positive ions producing the opposite effect: a decrease in CEBE with increasing species content. A thorough investigation of the induced CEBE shifts with species content is necessary to further correct C 1s XPS interpretation. This can be achieved using the methodology we have presented in this work.

## 7 Outlook

This work introduces a paradigm by which theory and experiment can be combined to generate low-energy atomistic structures with XPS predictions that conform to experimental spectra by design. This method reconciles experimental and simulated analysis of the structure of materials and, because of this, it appeals to both the computational scientist—who can have greater confidence in their results due to experimental agreement—and the experimentalist—who can obtain atomic-scale information about the structure and potential chemistry of their material.

By imposing agreement of simulated and experimental XPS data, new insights into the structure and composition of real materials can be gained without the inaccuracies apparent in experimental XPS deconvolution techniques, which can give an incorrect picture of a material’s constituents. Furthermore, one is not limited to using one experimental observable: although we have focused here on XPS, the approach can be readily extended to other analytical techniques. Multiple observables can be used simultaneously to elucidate specific experimental structures.

Prior approaches used empirical interatomic potentials and were limited to experimental observables that could be reproduced with simple analytical models. This work leverages the flexibility and predictive power of atomistic ML and paves the way for combining more complex computational models of experimental observables and *ab initio*-accurate MLPs to generate structures consistent with experiment.

## Supporting Information Available

Miscellaneous additional information, further tests, and benchmarks of the methodology and technical details of the calculations: (i) evolution of the XPS spectrum during a modified GCMC example run; (ii) difference in unoptimized and optimized GCMC runs for similar oxygen content; (iii) variation in modified GCMC runs with different σ; (iv) technical parameters of the DFT calculations; (v) correlation between CEBE error and local energy; and (vi) demonstration of the magnitude of *E*_pot_ and *E*_spectra_ variation (PDF)
