# Supplementary material for: Experiment-Driven Atomistic Materials Modeling: A Case Study Combining X-Ray Photoelectron Spectroscopy and Machine Learning Potentials to Infer the Structure of Oxygen-Rich Amorphous Carbon
Source: J Am Chem Soc. 2024 May 15;146(21):14645–59. doi: 10.1021/jacs.4c01897 (PMC11140750; doi:10.1021/jacs.4c01897)
Supplement: Supplementary file 1 — ja4c01897_si_001.pdf [file ja4c01897_si_001.pdf]

Supporting Information: Experiment-driven atomistic materials modeling: A case study combining X-ray photoelectron spectroscopy and machine learning potentials to infer the structure of oxygen-rich amorphous carbon

Tigany Zarrouk,[1,*] Rina Ibragimova,[1] Albert P. Bartók,[2,3] and Miguel A. Caro[1,†]

[1]*Department of Chemistry and Materials Science, Aalto University, 02150 Espoo, Finland*
[2]*Department of Physics, University of Warwick, Coventry CV4 7AL, United Kingdom*
[3]*Warwick Centre for Predictive Modelling, School of Engineering, University of Warwick, Coventry CV4 7AL, United Kingdom*

(Dated: 28 March 2024)

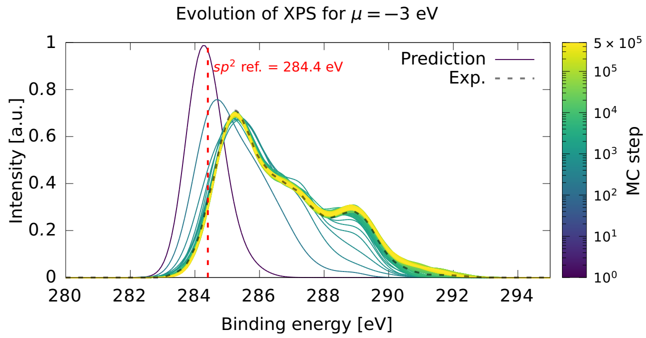

Figure S1. Evolution of XPS spectrum over the course of a single modified grand-canonical Monte Carlo run with optimization with respect to the high-oxygen content spectra at a chemical potential of $\mu = -3$ eV. The spectrum was sampled every 200 steps. The lines are colored according to the number of Monte Carlo steps.

I. EVOLUTION OF XPS DURING MODIFIED GRAND-CANONICAL MONTE CARLO

In Fig. S1 we show the evolution on a predicted XPS spectrum along a single GCMC run. We observe how the high-energy features, corresponding to C sites with one and, especially, two O neighbors, appear towards the end of the run.

II. DIFFERENCE IN UNOPTIMIZED AND OPTIMIZED GRAND-CANONICAL MONTE CARLO AT THE SAME OXYGEN CONTENT

In Fig. S2 we show, for similar oxygen content in the a-CO_x structure, that the XPS spectra of XPS-optimized and XPS-unoptimized runs look very different. In particular, the very high-energy features can only be reproduced when incorporating the XPS-based optimization.

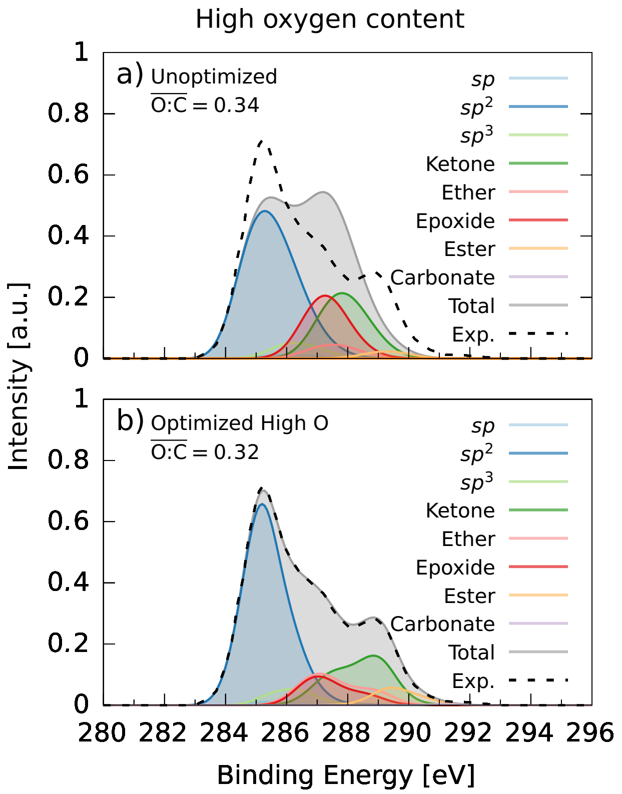

Figure S2. Comparison of XPS and deconvolution between unoptimized and optimized structures for the similar oxygen contents.

III. VARIATION IN MODIFIED GRAND-CANONICAL MONTE CARLO RUNS WITH DIFFERENT SIGMA

As briefly discussed in the paper and, in more detail, in Refs. [2–4], there are three main factors that affect the peak broadening in XPS fitting: instrumental, thermal and configurational (disorder). All of them affect experimental fitting while the last one, configurational broadening due to disorder, is accounted for in our method by explicitly generating an ensemble of atomic motifs (so that σ does not need to account for this). The in-

* tigany.zarrouk@aalto.fi
† mcaroba@gmail.com

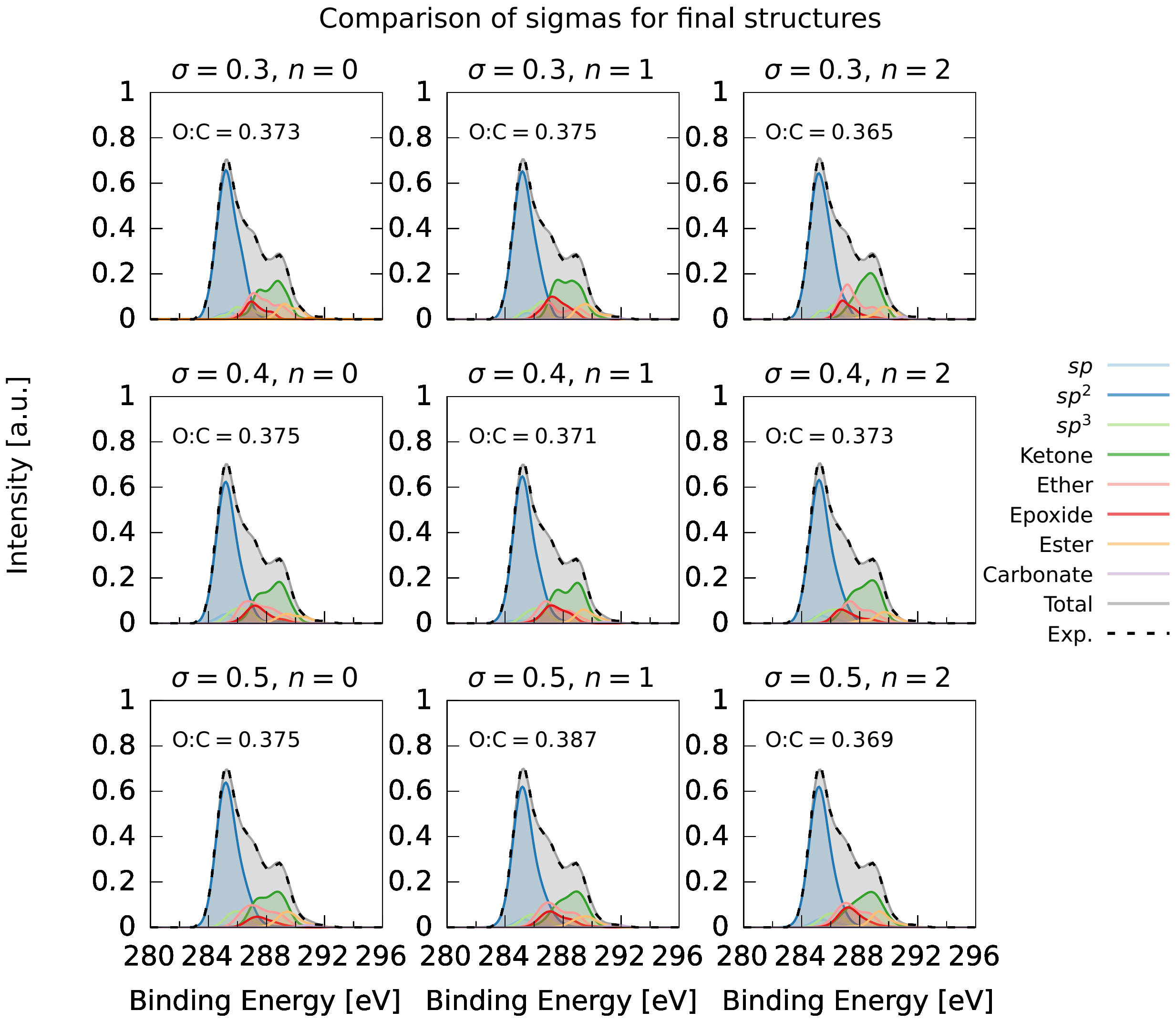

Figure S3. Deconvolutions of final structures resulting from modified grand-canonical Monte Carlo simulations for different sigma values. Three separate runs $n = 0, 1, 2$ were performed starting from the same initial pure amorphous carbon structure. The first, second and third rows correspond to $\sigma = 0.3$ eV, $\sigma = 0.4$ eV and $\sigma = 0.5$ eV, respectively. There is little variation in the final deconvolution results in the $\sigma = 0.3$–0.5 eV range.

strumental broadening is affected by the experimental setup. For instance, the broadening parameter for our method would differ when trying to reproduce an experimental XPS spectrum from synchrotron experiments (lower broadening because of the higher instrumental resolution) compared to lab-based measurements. Thus, in our approach, the broadening should be chosen based on physical grounds from the combined effect of instrumental and thermal broadening. We also note that the broadening is not "hard-coded" into our algorithm but can (and indeed should) be chosen by the user based on these physical considerations. In our case, we expect that the chosen value of 0.4 eV is appropriate for comparison to the experimental XPS results of Santini *et al.*

When running an optimization with a smaller smearing parameter, the expected result would be to obtain a more diverse set of structural motifs, so that the additional peak broadening required to fit the experimental spectrum is induced by structural disorder. Conversely, when the chosen smearing parameter is larger, a less diverse set of structural motifs can reproduce overlapping features in the target spectrum. Overall, for amorphous materials, we do not expect a big effect on the results from smearing parameters chosen within a reasonable range of parameters, which should be within the 0.3 eV to 0.5 eV range. This can be seen in Fig. S3. For materials with smaller intrinsic structural disorder (e.g., graphitic carbons), the effect might be more pronounced as the structural disorder is due to a comparatively small set of structural motifs. Thus, it is entirely appropriate and relevant to raise the issue of how the smearing should be chosen. We previously discussed the choice of smear-

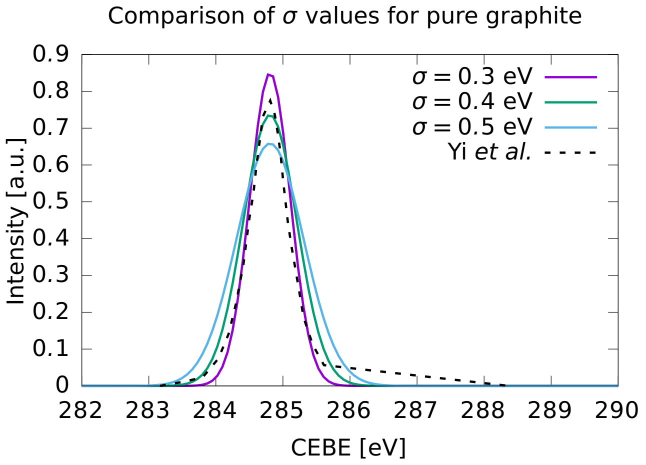

Figure S4. Comparison of simulated pure graphite spectra in comparison to experiment [1]. The predictions have been shifted to the maxima of the experimental peak. In pure graphite, there is no structural disorder, therefore the broadening is only due to thermal and instrumental effects. $\sigma = 0.3$–0.4 eV give a reasonable width when compared to the experimental data.

ing parameter based on physical grounds in more detail within the context of a different, but related, method to fit experimental XAS and XPS spectra in an two-part study [2, 3], where we concluded a value of 0.5 eV, *including the effect of disorder*, was appropriate for carbon materials. We further show here, when one has no structural disorder—e.g., for a pure graphite XPS spectrum, as found in the experimental work of Yi *et al.* [1]—that a smearing parameter of $\sigma \sim 0.3$ eV (or slightly bigger) is appropriate to account for thermal and instrumental broadening. See Fig. S4.

IV. TECHNICAL PARAMETERS OF THE DFT CALCULATIONS

The details of the density-functional theory (DFT) calculations are as follows. We used the Perdew-Burke-Ernzerhof [5] (PBE) functional and projector augmented-wave [6, 7] (PAW) pseudopotentials with $2s^2 2p^2$ and $2s^2 2p^4$ valence electron configuration for C and O, respectively (version `08Apr2002` of the VASP pseudopotentials library). The cutoff energy for the plane wave basis set was 650 eV, and reciprocal space was sampled with an automated Γ-centered k mesh generation with a minimal spacing between k points of 0.25 Å^{-1}. The contents of the VASP INCAR file used to generate the single-point DFT with the PBE exchange-correlation functional (PBE-DFT) training data are as follows:

```
PREC = Accurate
NSW = 0
ENCUT = 650
EDIFF = 1.0e-08
ISMEAR = 0; SIGMA = 0.1
ISIF = 2
KSPACING = 0.25
KGAMMA = .TRUE.
```

V. CORRELATION BETWEEN CEBE ERROR AND LOCAL ENERGY

The CO GAP includes two-body and core potential terms which improve its generality. This means that, even though the accuracy will be reduced in regions of configuration space where training data was not available, the MLP will remain stable and within reasonable bounds (it will not "blow up"). More rigorously, the accuracy is best for those configurations that contribute the most to the partition function's phase space integral, whereas those that should contribute little (those high in energy) will have worse accuracy but still contribute little to the partition function (they are still predicted to be high in energy). Thus, the properties derived from the potential energy landscape are not severely affected by extrapolation of the MLP. By contrast, our core-electron binding energy model uses solely a many-body descriptor and was trained on comparatively a lot less data. For this model, there is no guarantee that extrapolation (predictions far away from the training set, as can happen in MC) will lead to any "reasonable" predictions. In particular, the XPS prediction will only be accurate for low-energy configurations. Fortunately, this is not an issue within the context of the multiobjective optimization because those configurations with large CEBE error are discouraged from being accepted by the GCMC algorithm because they are high in energy, as we show below.

To provide more substantiated support for our argument, we computed error estimates for the CEBE model. We can do this easily because the CEBE model uses full GPR, and error estimates can be obtained in terms of how far from the training database a configuration is [8]; for the GAP, we do not estimate the error since the sparse GPR error estimate has extremely poor predictive power (we note that we could still estimate the GAP error using a committee model; however this would require a relatively large effort rewriting substantial parts of very sophisticated software, beyond the scope of this work).

As discussed, we expect that larger predicted CEBE errors should correlate with large local energies. As such, during modified grand-canonical Monte Carlo runs, trial configurations which give a large CEBE prediction error will be discouraged. This is demonstrated in Fig. S5, which shows the local energy for random configurations (C and O atoms which randomly populate a simulation cell) and molecular dynamics runs at a wide range of temperatures. We see there is a strong positive correlation of the CEBE prediction error and local energy. Past an appreciable CEBE prediction error, the local energy *exponentially* increases. These large local energies

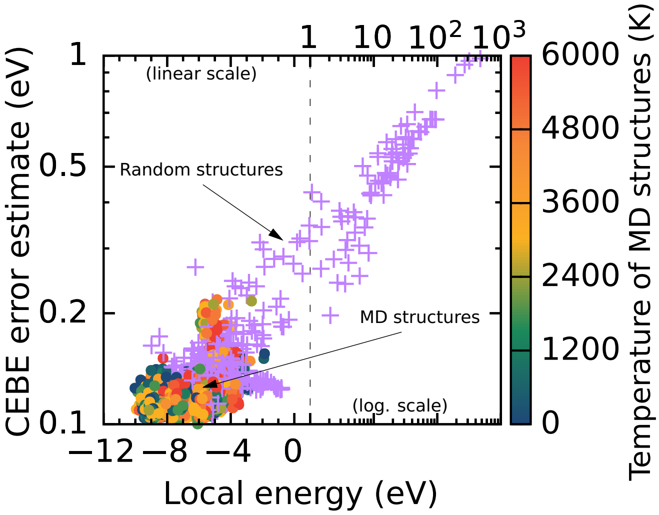

Figure S5. Variation in the error in the prediction of the core-electron binding energies (CEBEs) with the predicted local energy for different configurations. Purple crosses are random structures created by populating a simulation cell with a number of carbon and oxygen atoms at random positions. Colored points are configurations generated by NVT molecular dynamics simulations of amorphous carbon and oxygen systems at different temperatures, where the color bar shows the temperature at which they were run. The first half of the plot (from the left edge to the middle) has a linear-linear scale and the right half of the plot has a linear-log scale. Up to a local energy of 0 eV, there is a strong positive correlation of the CEBE error and the local energy. As the predicted CEBE error increases, the local energy *exponentially* increases. These increases in the local energy per atom inhibit configurations with a large CEBE error from being accepted.

discourage the acceptance of configurations with a large CEBE prediction error, thus minimizing the incidence of extrapolation errors on the results of the optimization.

VI. DEMONSTRATION OF THE MAGNITUDE OF E_{pot} AND E_{spectra} VARIATION.

Despite the large nominal values of the γ parameter, such values were necessary to obtain a good fit of the experimental spectrum without considerably inhibiting the relaxation of the structure. Once the dissimilarity between the predicted and experimental spectra had decreased to a sufficient level, the energy of the machine learning (ML) potential (MLP) dominated the dynamics. This is made clear by Fig. S6, were we see the variation in E_{pot} and E_{spectra} upon atomic displacement of an atom from a final configuration resultant from the modified grand-canonical Monte Carlo protocol, where the dissimilarity is small and comparable to that of 2×10^5 MC steps. We see that the change in the potential will generally be positive and greater in magnitude than that of the E_{spectra} term. The range of generated E_{pot} values are roughly twice that of the E_{spectra} term, showing that the E_{pot} term dominates the dynamics. Hence, one can conclude that the structure can relax well, and that E_{spectra} does not dominate the Monte Carlo procedure for sufficient simulation times, which was our aim when developing this method. Of course, some displacements will give configurations which will be accepted; these are configurations within the green region in the bottom left of the figure and compose a small fraction of the configurations generated.

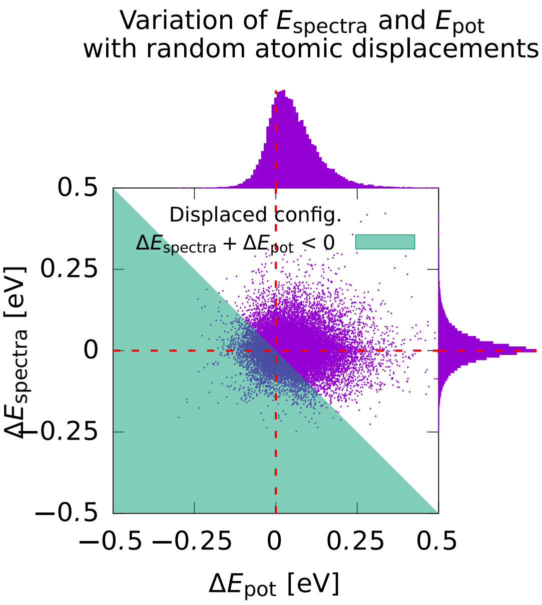

Figure S6. Variation of $\Delta E_{\text{spectra}}$ and ΔE_{pot} upon a random atomic displacement applied to a final structure ($\mu = -3$ eV, structure 0) resultant from our modified grand-canonical Monte Carlo approach. Each of the 20,000 points corresponds to the change in E_{spectra} and E_{pot} due to a single atomic displacement in a random direction. Displacement magnitudes were sampled from a uniform distribution from 0 Å to 0.1 Å. The green region denotes displacements in which the configuration would be accepted: where the total energy of the displaced configuration is lower than that of the original configuration. In general, the expected change in the potential is larger than in the E_{spectra} term, as such, E_{pot} dominates, despite the large nominal values of γ which compose the E_{spectra} term.

[1] Min Yi, Zhigang Shen, Xiaojing Zhang, and Shulin Ma, "Achieving concentrated graphene dispersions in water/acetone mixtures by the strategy of tailoring Hansen solubility parameters," Journal of Physics D: Applied

Physics **46**, 025301 (2012).

[2] A. Aarva, V. L. Deringer, S. Sainio, T. Laurila, and M. A. Caro, "Understanding X-ray spectroscopy of carbonaceous materials by combining experiments, density functional theory and machine learning. part I: fingerprint spectra," Chem. Mater. **31**, 9243 (2019).

[3] A. Aarva, V. L. Deringer, S. Sainio, T. Laurila, and M. A. Caro, "Understanding X-ray spectroscopy of carbonaceous materials by combining experiments, density functional theory and machine learning. part II: quantitative fitting of spectra," Chem. Mater. **31**, 9256 (2019).

[4] D. Golze, M. Hirvensalo, Hernández-León P., A. Aarva, J. Etula, T. Susi, P. Rinke, T. Laurila, and M. A. Caro, "Accurate computational prediction of core-electron binding energies in carbon-based materials: A machine-learning model combining DFT and GW," Chem. Mater. **34**, 6240 (2022).

[5] J. P. Perdew, K. Burke, and M. Ernzerhof, "Generalized gradient approximation made simple," Phys. Rev. Lett. **77**, 3865 (1996).

[6] P. E. Blöchl, "Projector augmented-wave method," Phys. Rev. B **50**, 17953 (1994).

[7] G. Kresse and D. Joubert, "From ultrasoft pseudopotentials to the projector augmented-wave method," Phys. Rev. B **59**, 1758 (1999).

[8] V. L. Deringer, A. P. Bartók, N. Bernstein, D. M. Wilkins, M. Ceriotti, and G. Csányi, "Gaussian process regression for materials and molecules," Chem. Rev. **121**, 10073 (2021).
